# Template-assisted synthesis of molecularly imprinted polymers for the removal of methyl red from aqueous media

**DOI:** 10.1186/s13065-023-00957-8

**Published:** 2023-05-11

**Authors:** Syed Rizwan Shafqat, Showkat Ahmad Bhawani, Salma Bakhtiar, Mohamad Nasir Mohamad Ibrahim, Syed Salman Shafqat

**Affiliations:** 1 Faculty of Resource Science and Technology, Universiti Malaysia Sarawak (UNIMAS), 94300 Kota Samarahan, Sarawak Malaysia; 2 Department of Chemistry, University of Sialkot, Sialkot, 51040 Pakistan; 3 Department of Chemistry, Pakistan Institute of Engineering and Applied Sciences, Nilore, Islamabad, Pakistan; 4 School of Chemical Sciences, Universiti Sains Malaysia, 11800 Penang, Malaysia; 5 Division of Science and Technology, Department of Chemistry, University of Education, Lahore, 54770 Pakistan

**Keywords:** Methyl red, Removal, Molecularly imprinted polymers, Precipitation polymerization, And aqueous media

## Abstract

This study entails the synthesis of molecularly imprinted polymers (MIPs) with good selectivity coefficients for azo dye as a potential sorbent material to extract azo dye from polluted aqueous media. A series of MIPs for methyl red (MR) as a template, were synthesized by changing the molar ratio of functional monomers, via precipitation polymerization format of non-covalent approach. Water-soluble functional monomer; acrylic acid (AA) was used to weave the frame work of polymers while ethylene glycol dimethacrylate (EGDMA) was utilized as crosslinking monomer. The impact of different experimental parameters, such as mole ratio of monomer (functional) to crosslinking monomer on the molecular recognition was investigated. The highly efficient and selective MR-MIP was used for the removal of spiked MR dye from different water samples. The selected imprinted polymer, MR1-MIP was able to selectively remove the MR molecules from aqueous media. A significant amount of dye was removed by MR1-MIP from the river water samples with a high degree of removal efficiency i.e. 92.25%. The imprinting factor of 3.75 for MR1-MIP indicated that the high selectivity in terms of adsorption for MR. A minimum loss of only ~ 3.35% in the removal efficiency within ten sequential cycles of adsorption–desorption study evidenced that MR-MIPs could be used as the most cost effective and best sorbent for the removal of MR from polluted water. Furthermore, the structural properties of MR-MIPs were characterized by FTIR and EDX, whereas TGA, SEM and BET were used to describe the thermal, morphological and surface structures of the particles, respectively.

**Graphical Abstract:**

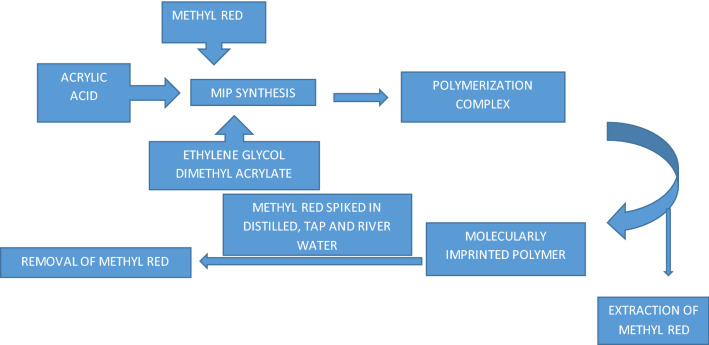

## Introduction

The color index reveals that the dying process is coupled with about 8000 commercial products [1, 2] and the use of synthetic dyes had been a significant part of nearly all industries. Dyes belonging to the recalcitrant class of pollutants and their color contamination can easily be identified [3]. Dye waste (10–15% of total dyes production) is a major part of a complex industrial sewage from these industries and could never be recommended for any household or industrial usage [4]. Paper and pulp industries [5], craft bleaching industries [6], tanneries [7], fabric industries [8], pharmaceutical industries, rubber, textile, leather, cosmetics and dyestuff manufacturing industries are amongst the source of generating immensely toxic colored effluents [9]. About 72% of the total dyes used and manufactured in the world are azo dyes [10] and 2/3 part of this is utilized in textile industries [11]. The inefficiency in textile processing of dying creates a huge amount of water stacked with azo dye stuff. These residues are directly transmitted into the water bodies and water with this unfavorable condition is potentially distressing for both toxicological and aesthetical reasons [12]. Azo dyes are capable of remaining persistent in the environment due to the presence of aromatic rings, azo and amino groups. The presence of dyes is undesirable in water even at very low concentrations. Due to their complex structure many of these are very difficult to degrade. Therefore, it is extremely necessary to remove the azo dyes from industrial waste before it is disposed into the water stream which may disrupt the aquatic biota [13]. Methyl red is a well-known dye being used in paper printing and textile dying sector. Potentially it is carcinogenic and also responsible for long term adverse effects in aquatic environment. It is toxic both by ingestion and inhalation and its contact may cause skin and eye irritation [14]. Therefore its removal owes population health and environmental protection.

Various biological and chemical methods have been reported in literature for the removal of azo dyes [15–24]. A wide range of physical techniques are also available for the elimination of dyes from contaminated aqueous media [25, 26]. Amongst all the employed approaches and the methods reported in the literature, adsorption has been proven as one of the most effective technique and the best equilibrium process [27]. Although the reported methods have satisfactory results for the removal of dyes but they lack specificity and have been used for the non-specific adsorption process which in turn results in poor selectivity. Therefore, a method which is highly specific and selective is required to remove dyes from the waste water effluents. Molecularly imprinted polymers (MIPs) are the solution for the selective removal of toxic materials such as dyes from waste water. MIPs are economical adsorbents/sorbents and offere a plenty of potential applications for future commercial purposes. MIPs have environmental safe procedure than any other current practices and can withstand harsh environmental conditions. MIPs are chemically, mechanically and thermally stable sorbents and are easily reproducible with extendable life cycles after their utilization [28, 29]. MIPs are highly cross-linked polymers with specific recognition ability for the target analyte. The advantages of MIPs as compared to the commonly used adsorbents are their high selectivity and specificity, reusability and low consumption. MIPs have many applications including solid-phase extraction [30] chromatographic separations [31], membrane separations [32], and sensors [33]. Previously MIPs have been applied for the removal/extraction of various analytes such as different dyes [34, 35], fungicides [36–38], melamine [39, 40], vanillic acid [41], gallic acid [42], cinnamic acid [43] piperine [44] and p-Coumaric acid [45] from different environmental and biological samples.

In this research, a series of novel molecularly imprinted polymers for methyl red (MR-MIPs) was synthesized to find out a suitable molar ratio of the components because it is the prime “key” to enhance the affinity of MIPs towards their analyte (MR) as well as responsible for increasing the number of imprinted polymer binding sites [43, 44] to best of our knowledge no such study is reported in literature for methyl red. For the first time in this study impact of porogenic solvent on pore size distribution or pore structure was investigated [46]. During the experiment, template/monomer ratios (mmol/mmol) were optimized for obtaining novel optimal synthetic conditions for MIPs. Versatile and incredibly resourceful acrylic acid (AA) was used as monomer (functional) while ethylene glycol dimethyl acrylate (EGDMA) was utilized as crosslinking monomer, no such study is reported for such a combination for synthesis of MIPs utilizing methyl red as template molecule. Toluene was used as a porogenic solvent. Precipitation polymerization protocol was endorsed and interaction between MR and AA has been established by non-covalent linkage. The chemistry of the template molecule, functional monomer and cross-linking monomer, nature of solvent, quantitative ratio of participants to each other and the temperature are all those parameters that could affect the affinity and efficiency of the MR-MIPs with analyte [47] therefore all these parameters were cleverly optimized in this study. The constant variables for MR-MIPs series in this experimental work were the template (quality/quantity) and temperature. The optimization of the binding efficiency can be enhanced by improving the synthesis methodology [48], therefore different ratios of template and functional monomer have been used to provide a good binding efficiency in the polymers. Since contact time of MIPs with the analyte/template (MR) during polymerization played an important role in producing better MIPs with higher efficiency therefore the polymerization conditions were optimized to obtained MIPs with best rebinding potentials.

## Materials and methods

### Materials

Methyl red (MR) and Congo red (CR) were purchased from Bendosen laboratory chemicals, Malaysia. Acrylic acid (AA), 2,2-azobisisobutyronitrile (AIBN) and ethylene glycol dimethyl acrylate (EGDMA) were obtained from Sigma-Aldrich, Germany. Toluene, acetic acid, acetone and methanol (MeOH) were received from R&M Chemicals, UK. All chemicals were used as received. The water samples used in the application process were retrieved from UNIMAS Soil Sciences Lab, UNIMAS water tank and from Samarahan River in Sarawak.

### Instruments/equipment

UV–Vis spectrophotometer (Model Perkin Elmer LAMBDA 25) was used to evaluate the concentration and absorbance of dye solutions. Infrared spectra (IR) of developed polymers were recorded on FTIR Model Thermo Scientific Nicolet Is10, before and after the complete wash off the template molecules. Scanning electron microscope (JEOL JSM 6930 LA model) typically integrated with energy-dispersive X-Ray analyzer (EDX/EDA/EDS) was used for the morphology and elemental analysis. The thermal properties of MR-MIP were characterized by using TGA Instrument, Universal Analyzer 2000 with Universal V4.7A software. For the direct measurements of surface area and pore size distributions of particles, nitrogen sorption–desorption porosimetry was done with Brunauer–Emmett Teller (BET) Quantachrome Autosorb.

### Synthesis of molecularly imprinted polymers

A series of MIPs were synthesized, for the first ratio 0.1 mmol (0.026 g) of MR as a template was dissolved in a reaction flask which contains 75 ml of toluene. After that, 1.00 mmol (68.5 µl) of AA was added to the mixture as a functional monomer followed by the addition of 16.00 mmol (2.97 ml) of EGDMA (cross-linking monomer). The insertion of an initiator in the mixture (0.030 g of AIBN) initiated free radical polymerization process. Therefore, the molar ratio of template, functional monomer and cross-linking monomer for MR1-MIP was 0.1:1:16, respectively. The entire mixture was sonicated for 10 min to homogenize the mixture. This was followed by purging of reaction mixture with Nitrogen gas (N2) for 15 min. Then the reaction flask was sealed and kept in a hot water bath at 60 °C for the first 2 h followed by 80 °C for the next 6 h. The produced polymer particles were then collected by using an assembly of vacuum filtration.

The same procedure was adopted for the synthesis of other two ratios with the only difference in the molar ratio of functional monomer. For the synthesis of MR2-MIP and MR3-MIP, 2.00 mmol (137.12 µl) and 3.00 mmol (205.68 µl) of AA were taken separately in conical flasks labeled “2” and “3” respectively. Hence, the molar ratios of template, functional monomer and cross-linking monomer for MR2-MIP and MR3-MIP were 0.1:2:16 and 0.1:3:16 respectively. NIP synthesis (control/reference material) had been performed on the same way but without the use of any template.

### Template extraction

The template was extracted from polymer particles by washing with MeOH and acetic acid (9:1, v/v) continuously until the template (methyl red) cannot be detected by UV–Vis spectrophotometer. After that the polymers were filtered to get “template free” MIPs. Finally, the polymer particles were dried in an open air for 24 h. The main purpose of template extraction was to produce cavities within the polymer which can actively rebind the template molecules from different solution media.

### Batch binding assay (rebinding assay)

The batch binding method was used to investigate the rebinding potential of MIPs, % removal efficiency and binding capacity/affinity [47] of MR-MIPs for methyl red. This also provides a way to choose a highly selective MR-MIP for target analyte from synthetic series of different compositions.

In this assay, a set of twelve conical flasks of 100 ml was washed, dried and labeled 1–12 for the evaluation of MR1-MIP. After that, 10 ml of 15 ppm standard solution of MR was added in each of the flask containing 0.1 g of the MR1-MIP. All the conical flasks containing polymer mixtures were agitated on an orbital shaker at 150 rpm for 6 h continuously and subsequently each of the labeled flasks was removed from the shaker at every 30 min time interval. Before filtration the mixture of polymer and solution in each conical flask was allowed to settle down to collect the supernatant for UV–Vis analysis. Binding assay for MR2-MIP, MR3-MIP and NIP was performed by using the same protocol as was adopted for MR1-MIP. Removal efficiency of methyl red by the MR-MIPs and NIP was determined by UV–Vis spectrophotometer. The λ_max_ of standard MR solutions during calibration experiments was obtained at 424 nm.

The following equations were used to calculate the removal efficiency (Q, mg/g) and adsorption capacity (Q_e_) of the samples:

1 $$ {\text{Removal}}\;{\text{efficiency}}\;{\text{Q}}\;\left( {\% {\text{R}}} \right) = \frac{{{\text{Co}} - {\text{Cf}}}}{{{\text{Co}}}} \times 100, $$

2 $$ {\text{Adsorption}}\;{\text{capacity}}\;{\text{Q}}_{{\text{e}}} = \frac{{\left( {{\text{Co}} - {\text{Cf}}} \right){\text{V}}}}{{\text{W}}}, $$

where Co is the initial concentration (mg/L); Cf is the final concentration (mg/L); V is the volume (L); W is the weight (mg) of polymer.

The MR-MIP with highest rebinding efficiency will be selected for further studies and tests on MIPs efficiencies determinations.

In order to characterize the total adsorption/sorption profile of selected MR-MIP, various parameters were studied and optimized for the uptake of MR from aqueous solutions using this selected polymer (Table 1). The parameters that were acquired for investigations are as follows: (i) MR concentration (ii) adsorbent dosage and (iii) pH.

**Table 1:** Adsorption parameters

S. no.	Parameters	Variation in parameter	Constant parameters
1	Different initial concentration	10, 15, 20, 25, 30 ppm	Agitation speed 150 rpm, contact time 90 min, adsorbent dose 200 mg, PH 7
2	Different polymer dosage	0.1, 0.2, 0.3, 0.4, 0.5 g	Agitation speed 150 rpm, contact time 90 min, PH 7, concentration 30 ppm
3	pH	5, 6, 7, 8, 9	Agitation speed 150 rpm, contact time 90 min, adsorbent dose 200 mg, and concentration 30 ppm

### Regeneration and repeated usage of MR-MIP

The regeneration of MIP was achieved by washing out the adsorbed dye by the mixture of MeOH: acetic acid (8:2, v/v). The washing of polymer was repeated continuously to ensure that the template (MR) has been completely removed from polymer matrix.

The stability and potential regeneration/reuse of the optimized MR-MIP sorbent were investigated. Any change in rebinding properties of MR-MIP was observed in ten sequential cycles of MR adsorption–desorption. Repeating application of adsorption–desorption was conducted under optimum conditions and then experimental results were obtained.

### Imprinting factor of optimized MR-MIP

Imprinting factor (IF) provides data to measure the strength of interaction of the imprinted polymer towards the target analyte. Whatever the conditions, IF is obviously greater than unity [48] which shows successful imprinting of polymers. The following equation was used to determine the IF.

3 $$ {\text{IF}}\;\left( \upalpha  \right) = \frac{{{\text{QeMIP}}}}{{{\text{QeNIP}}}}, $$

where QeMIP is the adsorption capacity of MR-MIP for MR; QeNIP is the adsorption capacity of NIP for MR.

### Selectivity test for optimized MR-MIP

No measurement is absolutely free from interferences. The degree to which a method is free from interference or contaminating agents in a matrix is considered to be precise. Affinity of MR-MIP towards the target analyte was examined prior to fabrication. In order to evaluate the selectivity of MR-MIP, the recognition experiment was executed in which the selection of methyl red (MR) was compared with Congo red (CR) as a structural analogous. Batch binding assay was used to test selectivity of the selected MR-MIP. MR-MIP along with its respective NIP was subjected to a selectivity test. A binary solution of 10 ml was prepared by mixing 5 ml of standard ~ 7 ppm MR solution with 5 ml of ~ 7 ppm CR standard solution for this study. This study was performed under optimum conditions.

The distribution ratios of MR between the MR-MIP and NIP were determined by following the Equation.

4 $$ {\text{Distribution}}\;{\text{ratio}},\;{\text{K}}_{{\text{D}}} = \frac{{\left( {{\text{Ci}} - {\text{Cf}}} \right){\text{V}} }}{{{\text{Cf}}\;{\text{m}}}}, $$

where Ci: the initial dye (MR or CR) concentration; Cf: the final dye (MR or CR) concentration; V: the volume of solvent used; m: the mass of MR-MIP/NIP used.

The selectivity coefficient for MR relative to binding competitor CR for MR-MIP and NIP was calculated as:

5 $$ {\text{K}}^{{{\text{sel}}}} = \frac{{{\text{KD}}\;{\text{Template }}\;\left( {{\text{Methyl}}\;{\text{red}} } \right) }}{{{\text{KD}}\;{\text{Interferent}}\;\left( {{\text{Congo}}\;{\text{red}}} \right)}}, $$

where K_D_ Template: distribution ratio of MR-MIP/NIP for MR; K_D_ Interferent: distribution ratio of MR-MIP/NIP for CR.

The relative selectivity coefficient (k′) was determined by the following equation as:

6 $$ {\text{K}^{\prime}} = \frac{{{\text{Ksel}} \;\left( {{\text{MR}}1 - {\text{MIP}}} \right)}}{{ {\text{Ksel}}\; \left( {{\text{NIP}}1} \right)}}. $$

The selectivity factor (β) for optimized MR-MIP was calculated by applying following equation:

7 $$\upbeta = \frac{{\upalpha \;{\text{template}} }}{{\upalpha \;{\text{interferent }}}}, $$

where α template_:_ Imprinting factor towards MR; α Interferent: Imprinting factor towards CR.

### Applications of optimized MR-MIP in different aqueous media

In order to determine the successive confirmation of designed MR-MIPs, rebinding process was employed in real world samples. The optimized protocol was applied to water samples (distilled water, tap water and river water) in order to measure the ability of selected MR-MIP to specifically extract MR. The water samples availed in this practice were retrieved from Samarahan river-side in Sarawak and UNIMAS soil sciences lab. Filtration and centrifugation procedure were adopted to eliminate the residues prior any extraction/removal by optimized MR-MIP and then stored in research laboratory for further usage. The experiments on optimized parameters were conducted thrice to obtain the average results and observe the deviations in the results. In this study, the selected MR-MIP and its respective NIP were used for the extraction/removal of MR from spiked water samples to measure the effectiveness of MR-MIP compared with that of NIP.

## Results and discussion

### Synthesis of molecularly imprinted polymers for methyl red

**Fig. 1:**
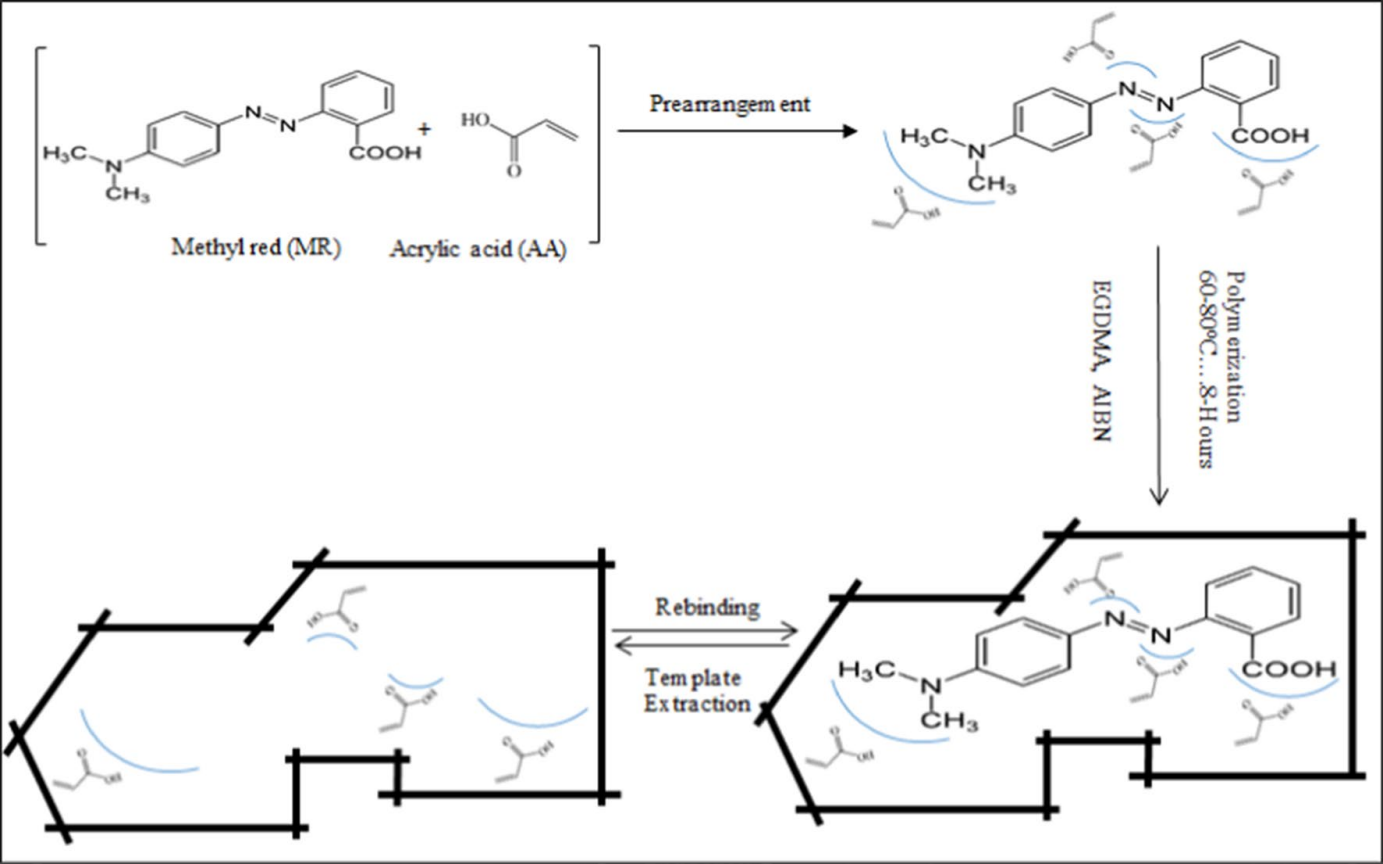
Schematic representation for the synthesis of MR-MIPs with MR as a template, AA as a functional monomer, EGDMA as a cross-linking monomer and AIBN as an initiator

Figure 1 depicts a schematic illustration of the synthesis of MR-MIPs. It can be seen in the reported investigations that molecular imprinting was done on relatively tiny molecules with a low polarity and a small number of ionizable groups [49–52]. As imprinting effect depends upon the size of template [53] hence in this work, a polar template, MR of medium molar mass (269.3 g/mol) containing one ionizable functional group (carboxylic group –COOH) was studied. A non-covalent approach was employed to develop imprinted polymer particles with well orientated MR molecules. The development of MR-MIP particles was conducted in three steps:

i. Formation of template monomer complex
ii. Polymerization and
iii. Extraction of template.

Weak forces may play a role in the creation of the template-monomer complex in the porogenic solvent prior to polymerization in molecular imprinting. The choice of functional monomer and absolute amounts of polymerization components can aid in the creation of MIPs with strong imprinting and extraction abilities. They also influence the stability of the developed polymer and thus the execution of the MIPs to interface mainly with the objective analyte [29]. An excess of functional monomer compared to template can favor the prepolymerization template-monomer complex so, when the MR was allowed to stir with AA in porogenic solvent, the electrostatic and non-electrostatic forces were active, establishing non-covalent interaction between the MR and AA molecules prior to polymerization [54–56]. The H-bonding was the primary driving force for the molecular recognition between AA and MR (target molecule). Carboxyl group (–COOH) of AA made it easier for it to create H-bonds with the amino group (tertiary amine) and carboxyl group of MR molecule. Azo groups (N=N) and HC– sites of aromatic rings may also be available for interactions in template molecule that may contribute to the fabrication of MR-MIP. As long as the mechanical stability and morphology of the polymer are concerned, cross-linking monomer is used that maintains the recognition sites [54]. EGDMA was particular for cross-linking. The crosslinking by EGDMA produced a strong backbone of the MR-MIP. The assigned porogenic solvent, toluene attracted the solubilization of the constituents and responsive in the AA–MR interactions thus control and optimize the distribution of imprinting cavities within the resulted MR-MIP. Toluene is a non-polar aprotic solvent that ensures good solubility of the template and contributes to the formation of the H-bonding between the template and functional monomer [57, 58]. The selection of porogenic solvents and their optimal amount can influence the selectivity, porosity and the surface areas of MIPs [59]. All the synthesized MR-MIPs and NIP were obtained in powdered form with dark red and off white appearance, respectively. An imprinted polymer matrix was generated by leaching off the MR molecules which left the impressions complementary to the shape and structure of MR molecules thus can actively rebind MR.

### Batch binding assay for MR-MIPs

**Fig. 2:**
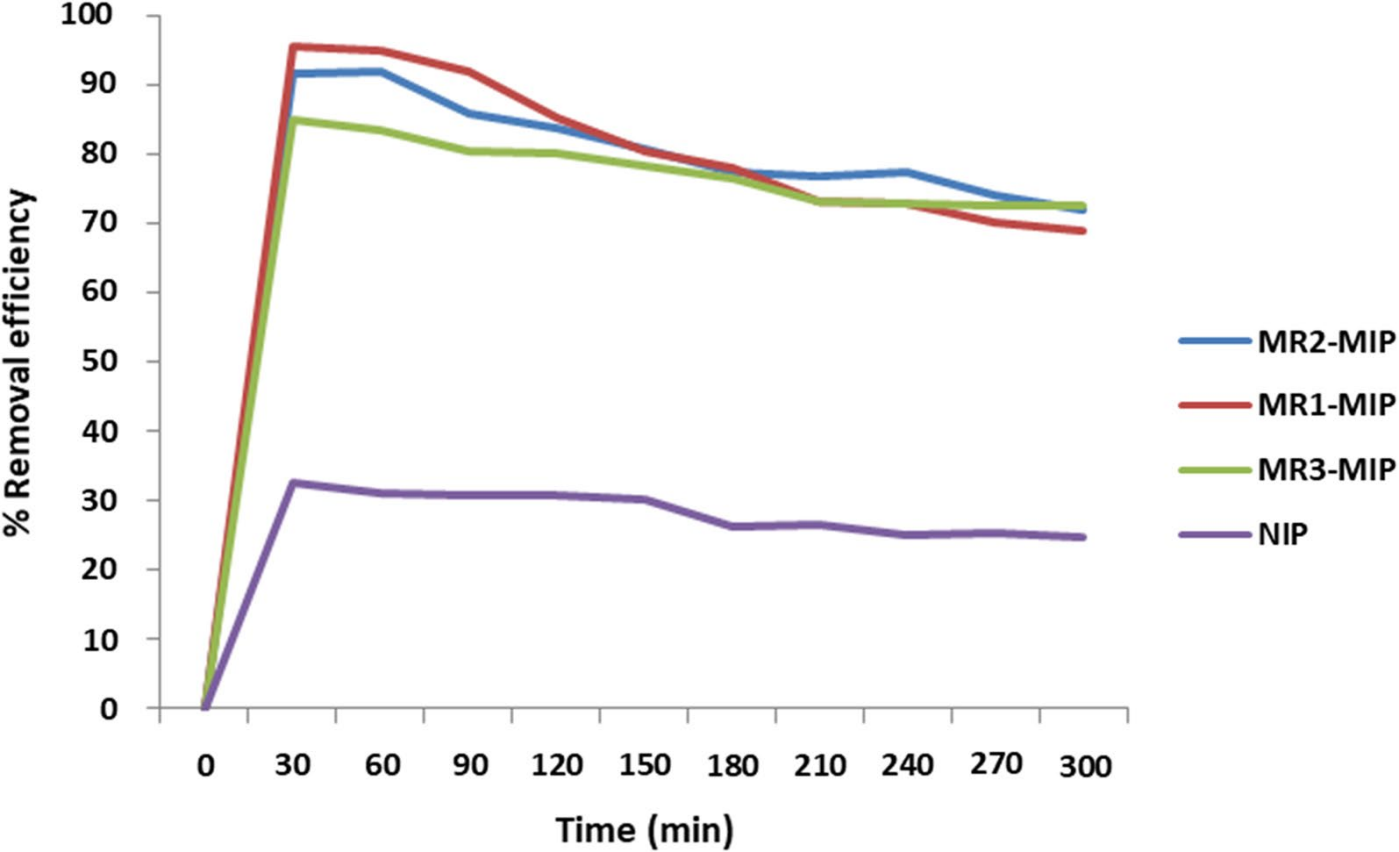
Batch binding assay for MR-MIPs with varying mole ratio of functional monomer

The amount of molecular recognition of produced MR-MIPs was measured using a batch binding experiment. The spatial structure (three-dimensional) of the template molecule and the degree of matching of the binding sites were the major determinants of molecular recognition [29, 60]. The removal efficiency (%R) and binding capacity (Qe) of MIPs are crucial that signifies the effectiveness of the synthesized MR-MIPs in binding the analyte. The highest binding efficiency amongst MR-MIPs series (MR1-MIP, MR2-MIP and MR3-MIP) developed by changing the mole ratio of functional monomers and was for MR1-MIP (90.75%). In relation to the three different ratios of MR-MIPs, 0.1:1:16 (MR1-MIP) molar ratios of template, functional monomer and cross-linking monomer exhibited the highest removal efficiency. The lowest performance was by NIP because of the weakest recognition sites due to the absence of template during the synthesis that inhibits the bindings. Figure 2 portrays the removal efficiencies (%R) of developed MR-MIPs and NIP.

The strength of binding efficiencies for the MR-MIPs series is arrayed as follows:

**Table:** 

NIP-1″ (0.1:1:16)<	MR3-MIP (0.1:3:16)<	MR2-MIP (0.1:2:16)<	MR1-MIP (0.1:1:16)
27.03%	85.63%	86.42%	90.75%

The improved efficiency of MR1-MIP was due to its compatible template, functional monomer, and cross-linking monomer ratios, which may have more specific recognition sites for the MR than the other two MR-MIP ratios. The affinity of MIP became more non-specific when the ratio of functional monomer to template was increased [61]. This could explain why MR2-MIP and MR3-MIP had a lower binding affinity for their respective analyte (MR) and therefore had poor removal effectiveness. In both the second and third ratios of MR-MIPs, an excess number of functional monomers (AA) causes’ self-association [63] of functional monomer molecules within themselves, resulting in a decrease in the creation of binding sites on the respective MR-MIP. The choice of solvent is also a very important factor. In this study, toluene was used as the porogenic solvent for MR-MIPs synthesis. Being aprotic solvent, toluene did not interfere with the H-bond formation between the participants rather it facilitated and made the polymers porous [59, 62].

As control polymers, NIP was developed and selected based on the best ratio compositions and this step is called the base extraction [63, 64]. The adsorptive response of corresponding NIP was very low and linear over the full time range of measurements because NIP exhibits the weakest recognition sites available for template molecules.

The impact of contact time on the adsorption of MR by MR-MIPs can be observed in the batch binding assay section. Based on the results obtained, the maximum extraction/removal of MR dye at 30 min had been used in the subsequent studies for MR1-MIP. It is clearly shown in Fig. 2 that the rate of adsorption is initially very high, approaches a dynamic equilibrium and then remains almost constant despite further increase in contact time for MR-MIPs. It is clearly depicted in the graph (Fig. 2) that with the increase in the contact time, the removal efficiency of MR-MIPs also increases. Initially, the dye molecules rapidly move towards the MR-MIPs as the MIPs are starved for these dye molecules and the number of binding sites is very large that allow adsorption/sorption to take place very easily. As the time increases, the removal efficiency becomes constant without any further variations. At that time, the quantity of dye molecules adsorbed by the MR-MIPs and the amount of dye molecules desorbed from the polymer have reached a condition of dynamic equilibrium. So, the binding sites of MR-MIPs became saturated and do not permit any further adsorption/sorption to proceed [55, 65–68]. The removal efficiency of MR1-MIP is increasing up to certain time period and then becomes almost constant at later stages due to the saturation of binding sites. The concentration of dye solutions may not change considerably after 30 min for MR1-MIP. It is primarily assumed that the active binding sites of MR1-MIP had been fully saturated very early and the diffused pores did not permit the adsorption process to continue anymore. The maximum removal efficiency of 90.75% for MR1-MIP was observed at 30 min. Hence, the contact time of 30 min was optimized for MR1-MIP as a representative of the MR-MIPs series.

### Effect of MR concentrations on the uptake behavior of MR1-MIP

**Fig. 3:**
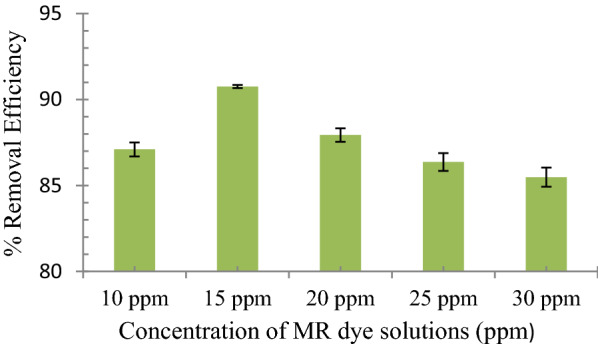
Effect of MR initial concentrations on the uptake behavior of MR1-MIP

The concentration of dye has an outward effect on its expulsion from any fluid media by MIP. The removal efficiency (%R) of MIP was enhanced with the increase in the initial concentration of dye (Fig. 3). An increase in the initial dye concentration refers to the increase in dye molecules. The active binding sites of the MIP are well surrounded by dye molecules, resulting in greater and more effective adsorption. This trend can also be attributed to the fact that when the dye concentration rises, the responsible driving factors for mass transfer increases. The selected MR1-MIP presented similar trends as shown in Fig. 3. The increasing trend of adsorption by MR1-MIP was observed up to a certain level of dye concentration. Then, further increases in the dye concentration have no apparent change in the adsorption process. This means that all the available binding sites on the polymer particles have been fully saturated [55, 65–68]. The maximum removal efficiency (%R) for MR1-MIP was found to be 90.75% with 15 ppm of the MR solution. Principally, 15 ppm was considered as the optimum concentration for MR1-MIP form calibration experiments.

### Effect of polymer dosage on the uptake behavior of MR1-MIP

**Fig. 4:**
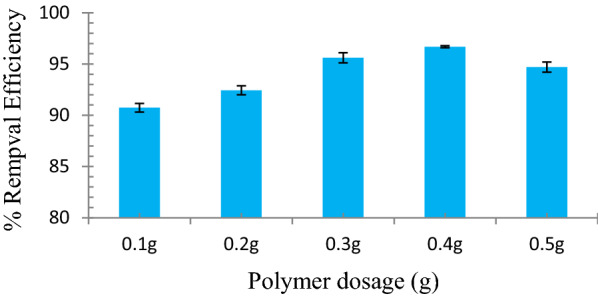
Effect of polymer dosage on the uptake behavior of MR1-MIP

In this research, the contact time and the concentration of MR solutions were kept constant but the dosage of MR1-MIP was studied using different amounts of polymer. The effect of polymer sample dosages (MR1-MIP) on the amount of MR removal was expressed as %R. Figure 4 shows that the removal efficiency (%R) of dye increases for MR1-MIP, with the increase in the polymer dosage up to a certain limit which was followed by a gradual decline. Graphical depiction (Fig. 4) can be detailed out that availability of binding sites of adsorbent has been increased by topping up the polymer dosage. It is feasible for MR dye molecules to spread over the MR1-MIP, resulting in an increase in adsorption phenomenon. Conversely, with the increase in dosage amount, the polymer particles formed aggregates and due to this aggregation the number of available binding sites accessible to dye molecules have decreased [55, 65–68]. The maximum %R for MR1-MIP was observed at 96.68% with an adsorbent dosage of 0.4 g. Thus, a dosage of 0.4 g was considered as optimum dosage for the selected MR1-MIP at optimum agitation time and optimum concentration [69, 70].

### Effect of MR solution pH on the uptake behavior of MR1-MIP

**Fig. 5:**
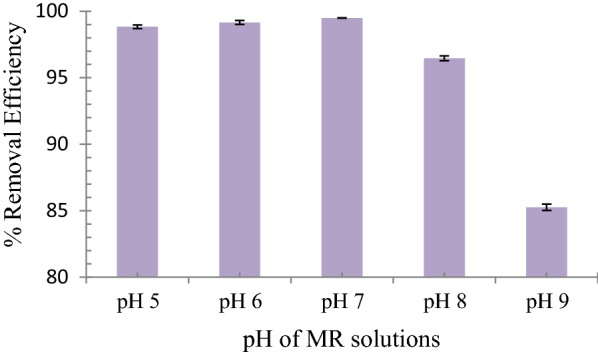
Effect of MR solution pH on the uptake behavior of MR1-MIP

The change of solution pH was conducted to know if MR-MIPs were pH sensitive polymers that exhibit conformational changes which may lead to swelling or shrinking behavior of the imprinted polymers [71]. Both the adsorbent surface properties and the degree of ionization of dye molecules are induced by altering the pH of solution. It is evident from the results in Fig. 5 that the highest removal efficiency (%R) of MR was achieved at pH 7 with a %R of 99.51% for MR1-MIP. Because there are no structural changes in dye and MIP configurations at optimum pH (pH 7), maximal dye uptake was found as compared to acidic or basic media. The neutral (pH 7) condition was shown to be the best for the most interactions between the template and the imprinted sites. The specific binding sites bearing –COOH groups on the dye molecule might have changed due to the alteration of pH and consequently decreases possibility of binding with the polymer. There is a huge amount of H+ ions in an acidic media acting as competitor, the hydrophobic interactions are increased and H-bonding between MR molecules and selective binding sites are decreased and results in lowering the removal efficiency of MR1-MIP. While in basic media (higher pH), the low removal efficiency may be due to hydroxyl ionic interpretation of MR.

According to Chen [72] the binding energy of the selectivity sites in MIPs is decreased in the non-optimum conditions of time, dye concentration, dosage of the polymer and pH of solution. As non-optimum conditions affected the adsorption pattern, the selected MR1-MIP was optimized in a succession and in conclusion to the extraction/removal efficiencies of the optimized MR1-MIP were summarized in Table 2.

**Table 2:** Study of process parameters on the adsorption/sorption by selected MR1-MIP

Molecularly imprinted polymer	Process parameters				
	Agitation/ contact time	Concentration	Dosage	pH	IF (α)
MR1-MIP	30 min	15 ppm	0.4 g	7	${\text{IF}}\;\left( \upalpha \right) = \frac{{{\text{Q}}\;{\mathbf{MRI}} - {\mathbf{MIP}}}}{{{\text{Q}}\;{\text{NIP}} - 1}}$
	Q = 90.75%	Q = 90.75%	Q = 96.68%	Q = 99.51%	3.752
	Qe = 107.0	Qe = 107.0	Qe = 28.80	Qe = 30.04	

### Imprinting factor of optimized MR1-MIP

Definite recognition properties of MR1-MIP for MR template with respect to its NIP were established in the form of imprinting factors (IF, α). An imprinting factor (IF, α) corresponded to the ratio of the template (MR) bound to the MR1-MIP versus the template (MR) bound to the NIP. It showed a positive correlation of the interaction strength. In terms of a high IF value, it should have a strong interaction with the template molecule, resulting in higher adsorption capacities (Qe) than non-imprinted polymers. IF (α) value for the MR1-MIP is display in Table 2. Higher IF value (α) of 3.75 for MR1-MIP indicated major degrees of imprinting in it. This significant IF in MR1-MIP may also attribute to the usage of appropriate medium polar solvent. It means that medium polar solvents are good porogenic solvent compositions for preparing MIPs for the azo dyes [53].

### Repeated use of optimized MR1-MIP

A substantial benefit of MIPs is that their “ability of recognition” can be restored after successive use in the binding. MIPs can be engaged several times to adsorb the target material and supposed to be no change in their “recognition ability”. There was a very slight variation in MR rebinding by MR1-MIP in adsorption–desorption cycles. MR1-MIP was steady enough for the adsorption–desorption cycles without a noticeable decline in the removal efficiency (%R) for MR. Table 3 illustrates that decline in MR rebinding between first and tenth cycle was ~ 3.35% for MR1-MIP. A slight variation in “recognition ability” and adsorption for MR explained the outstanding stability of MIP [56].

**Table 3:** Effect of reused times on the % R of optimized MR1-MIP

Adsorption–desorption cycle	% R (Q) for MR by MR1-MIP (%)
Cycle-1	99.50
Cycle-2	98.52
Cycle-3	98.43
Cycle-4	98.40
Cycle-5	98.01
Cycle-6	98.01
Cycle-7	98.50
Cycle-8	98.32
Cycle-9	98.06
Cycle-10	97.20
Overall loss after 10 cycles	3.351

### Selectivity test for optimized MR1-MIP

Sensing property of MR1-MIP for MR was evaluated by employing the selectivity test. Congo red (CR) was selected as possible interfering and competitive agent for MR. Both the dyes are anionic azo dyes and are structurally analogous to each other. Most of the physical and chemical properties exhibited by them are also comparable. The data was derived by employing UV–Vis spectrophotometric analysis and then computed by using standard equations for distribution ratios (K_D_), selectivity coefficients (K^sel^), relative selectivity coefficients (k′) and selectivity factors (β) as shown in Table 4. When monomers and templates are used in the proper ratios during the pre-polymerization process, several high-affinity sites will emerge, which may change the template distribution ratios in MIPs. The distribution ratios can be described in a variety of ways, for as by comparing the adsorption concentrations of the template and the interferent with an identical solutions initial concentration. The distribution coefficient D is calculated from their ratio. Selectivity is measured by the ratio qA/qB = DA/DB. The results in the given table (Table 4) revealed that distribution ratios of MR in MR1-MIP were noticeably higher than the distribution ratios of its competitor (CR). Outstanding selectivity coefficient value indicates MR1-MIP is very particular for MR dye removal and the imprinting technique was very effective. Molecular recognition phenomenon may well be comprehended by the selectivity trial of the MR1-MIP. Both MR and CR are structurally in agreement with each other but dissimilar too, the spatial diameter of MR is relatively small than that of CR. There is one carboxylic functional group and one –N (CH_3_)_2_ groups in MR, but there are two carboxylic functional groups and two –HN_2_ groups in CR [67, 73]. The studies declared that functional groups of MR (template) located at the edges of molecules might have favourable interaction with the active binding sites of MR1-MIP and was easily entrapped into the cavities rather than CR (competitor) [73]. MR1-MIP performed well as it was more selective towards its template (MR). The higher distribution ratio of MR is also due to the fact that MR1-MIP can recognize and attach the MR molecules by specific binding sites that have been preserved as a memory [54, 73]. Template (MR) molecules can easily attach to the relatively matched cavities in size and shape, while an interferent can bind poorly due to nonspecific interactions [74]. Moreover, template molecules and interferent have their higher distributions in the MR1-MIP than NIP because NIP is lacking of binding sites.

**Table 4:** The distribution ratio, selectivity coefficient, relative selectivity coefficient andselectivity factor for optimized MR1-MIP and NIP

Imprinted polymer	Target	K_D_ (MIP)	K_D_ (NIP)	K^sel^ (MIP)	K^sel^ (NIP)	K″	β
MR1-MIP	Template (MR)	~ 19.12	4.312	3.941	1.264	3.117	3.091
	Interferent (CR)	4.851	~ 3.411				

### FTIR analysis for MR-MIPs

**Fig. 6:**
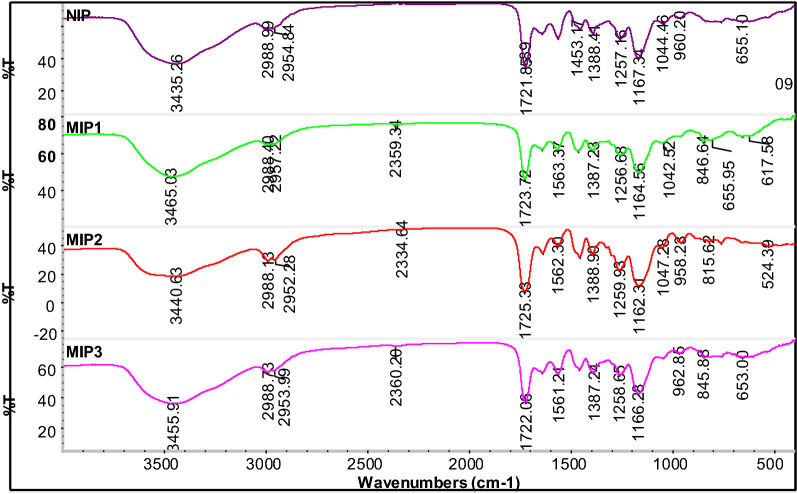
FTIR spectra of MR-MIPs synthesized by changing the mole ratio of functional monomers, before leaching off template

**Fig. 7:**
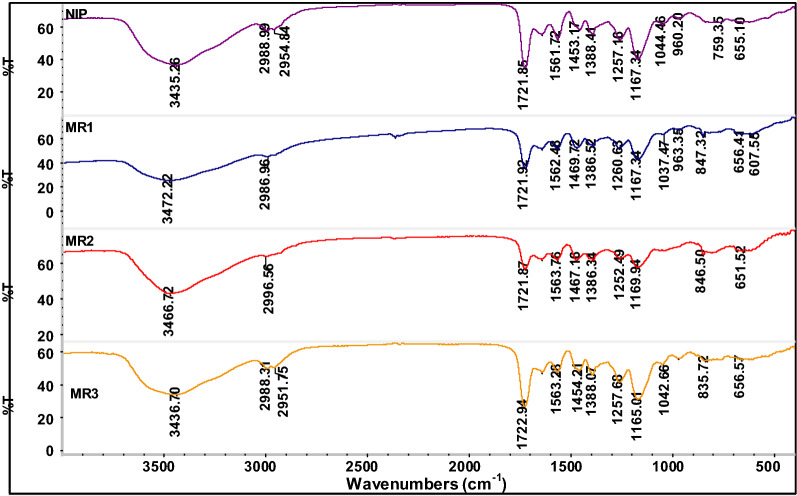
FTIR spectra of MR-MIPs synthesized by changing the mole ratio of functional monomers, after leaching off template

FTIR analysis was performed to make sure the interactions between MR, AA and EGDMA. This analysis is very critical to determine the chemical characteristics and the composition of imprinted polymers. Different ratios of EGDMA cross linked MR-MIPs that have non-covalently entrapped the MR molecules in their network were run for FTIR analysis, before and after leaching off the template. There was observed a very slight difference in the peak positions and their intensities for the FTIR spectra of different MR-MIP ratios. However, FTIR spectra of unleached MR-MIPs, leached MR-MIPs and NIP, respectively, presented a very closeness in the location of specific peaks, including those characteristic peaks that credited the backbone structure of these developed polymers [75]. The FTIR spectra of EGDMA cross linked MR-MIPs and the respective NIP were recorded in the region of 4000–500 cm^−1^. Figure 6 is furnished with FTIR spectra of unleached MR-MIPs synthesized by changing the mole ratio of functional monomer. The two remarkable peaks in functional group region at 1730 cm^−1^ (C=O stretching) and 1160 cm^−1^ (C–O stretching) in these spectra, support and ascribe the existence of EGDMA as a crosslinking monomer in the developed polymers. Also several peaks in the finger print region between 1600 and 1000 cm^−1^ revealed the EGDMA presence e.g. peaks at 1452 cm^−1^ and 1160 cm^−1^ correspond to –CH_2_ scissoring and symmetric/antisymmetric stretching for –O–R respectively of EGDMA. The H-bonding that involves –COOH group of AA and MR can be translated into the stretching of –OH group and refers to the completion of polymerization and entrapping of dye molecules. Carboxylic acid –OH stretching bands in the spectra of all unleached MR-MIPs (Fig. 7) at 3564 cm^−1^ and 1157 cm^−1^ were broader and more intense than that of free –OH vibrations that indicated the involvement in an intermolecular H-bonding. After the successful leaching off template, a comparison of the spectra was drawn for these MR-MIPs and the corresponding NIP. The absence of template bands and shifting of some peaks in Fig. 7 is an indication for the effective removal of the dye from the imprinted polymers. Characteristic peak positions in all the spectra attributed the same chemical composition of all the synthesized polymers during polymerization. According to Brune and Schink [76], the peak of –OH group from AA after the leaching off the template was identified due to the absence of H-bond disruption. Carboxylic acid –OH stretching bands in all leached MR-MIPs were shifted to about 3454 cm^−1^ and 1157 cm^−1^. According to the spectra in Fig. 7, one can see that all the peaks present in the MR-MIPs are also present in the NIP. However, the intensity of the peaks varies as compared to the intensity of the peaks present in the spectra of MR-MIPs before leaching and after leaching off the template. The intensity of the peaks is higher in the case of MR-MIPs before washing which is owing to the presence of MR dye while the intensity of the peaks is low in washed MR-MIPs due to the removal of MR. However; intensity of the peaks is very low in the case of NIP. As the intensity is a relative term and depends on the concentration, the FTIR analysis has significantly proven the successful synthesis of MR-MIPs [77].

### EDX analysis for selected MR1-MIP

**Fig. 8:**
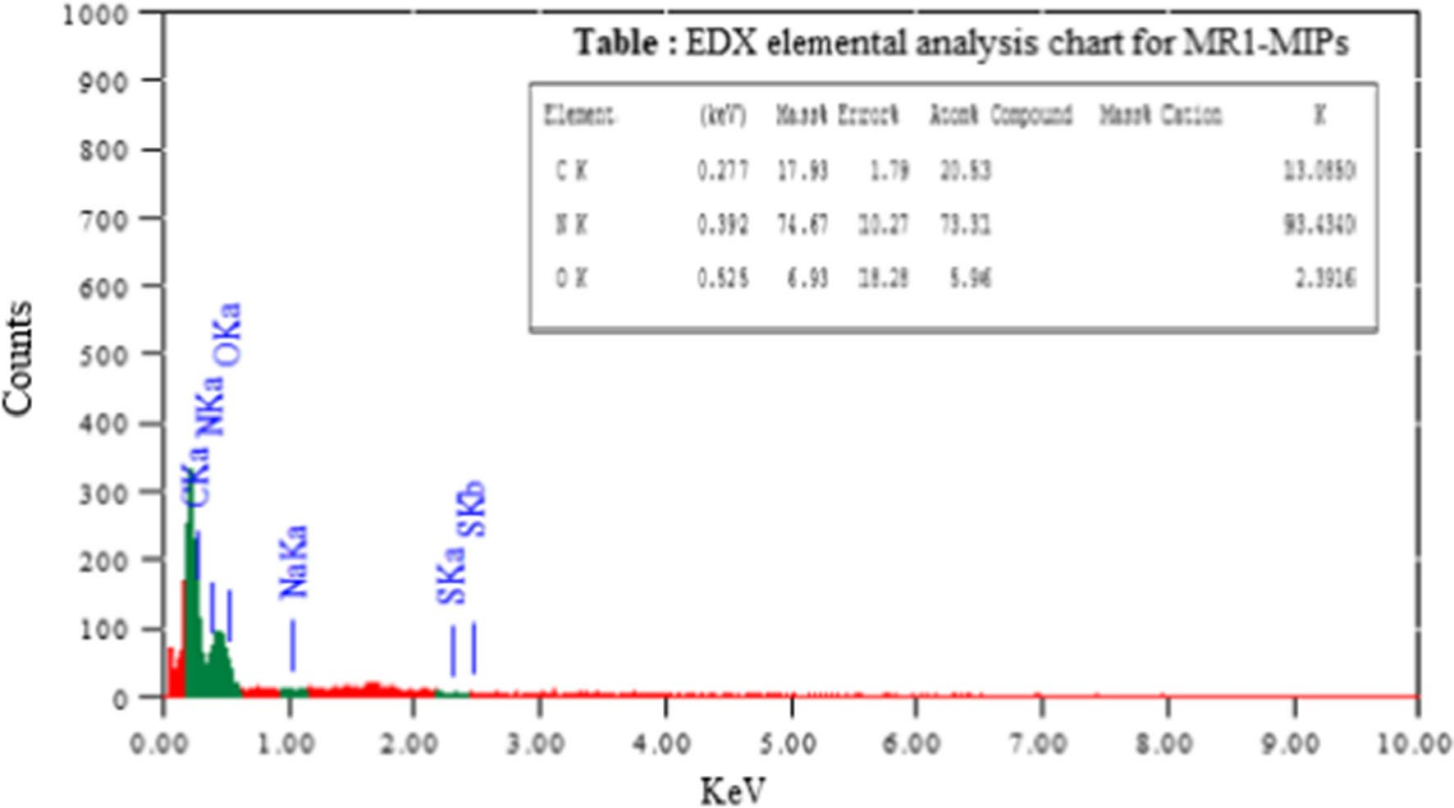
EDX spectrum and elemental analysis chart for MR1-MIP

The presence of every single constituent of the participator (template, functional monomer and cross-linking monomer) in the polymerization process was evaluated by EDX [78]. MR1-MIP was dried and assessed without the leaching off template (MR). The percent (mass % and atom %) abundance of all components in the backbone structures of MR1-MIP are depicted in the inset of Fig. 8. MR1-MIP polymer particles are composed of carbon, nitrogen and oxygen. The spectrum revealed that the nitrogen might be from MR while carbon and oxygen were from AA and EGDMA as well as from the dye molecules.

### TGA analysis for selected MR1-MIP

**Fig. 9:**
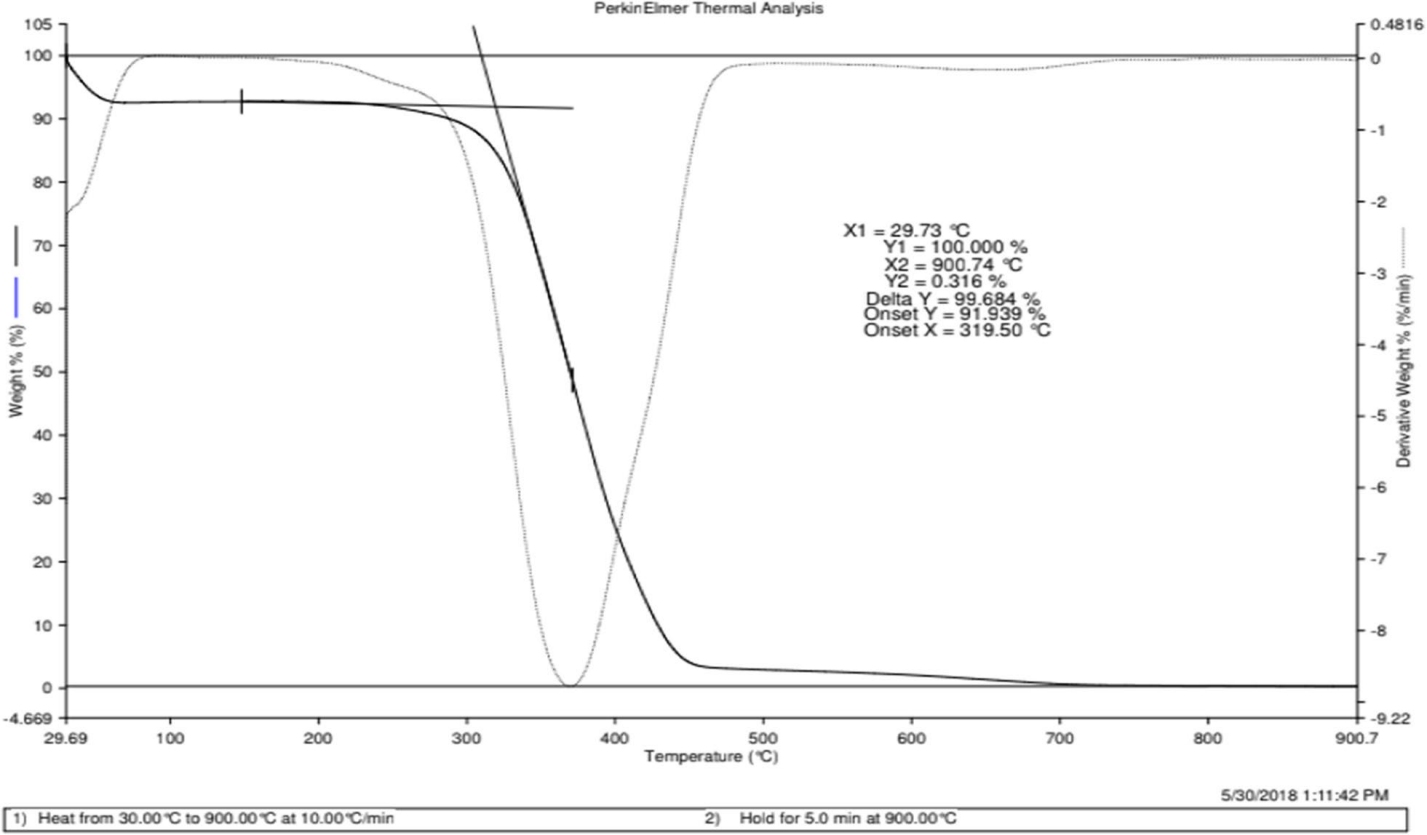
TGA spectrum of methyl red molecularly imprinted polymer MR1-MIP

TGA is a thermal analysis technique which is used to characterize the properties like thermal stability and mass loss of materials. The thermal degradation behavior of EGDMA cross linked poly (AA) MR1-MIP was studied in the range of 30–900 °C. The TGA spectrum that represents mass loss and the thermal stability of EGDMA cross linked poly (AA) MR1-MIP is shown in Fig. 9. Thermal analysis data of analyte depends on its molecular weight, polymeric architecture, and synthetic route and moisture contents [79]. The spectrum showed weight loss at two stages. About 8% weight losses by MR1-MIP were obtained at the first stage which was due to the removal of water (free and bound) from the imprinted polymers. The second stage of weight loss was observed in the range of 270–550 °C. This 90% weight loss of cross linked MR1-MIP might be due to the degradation of poly (AA) backbone. The decomposition at a higher temperature showed the greater thermal stability of highly cross linked poly (AA) MR1-MIP. Furthermore, the DTA curve in the spectrum also strongly supports the phenomena of polymer decomposition observed in TGA.

### Morphological study of MR1-MIP/NIP1 by using SEM

**Fig. 10:**
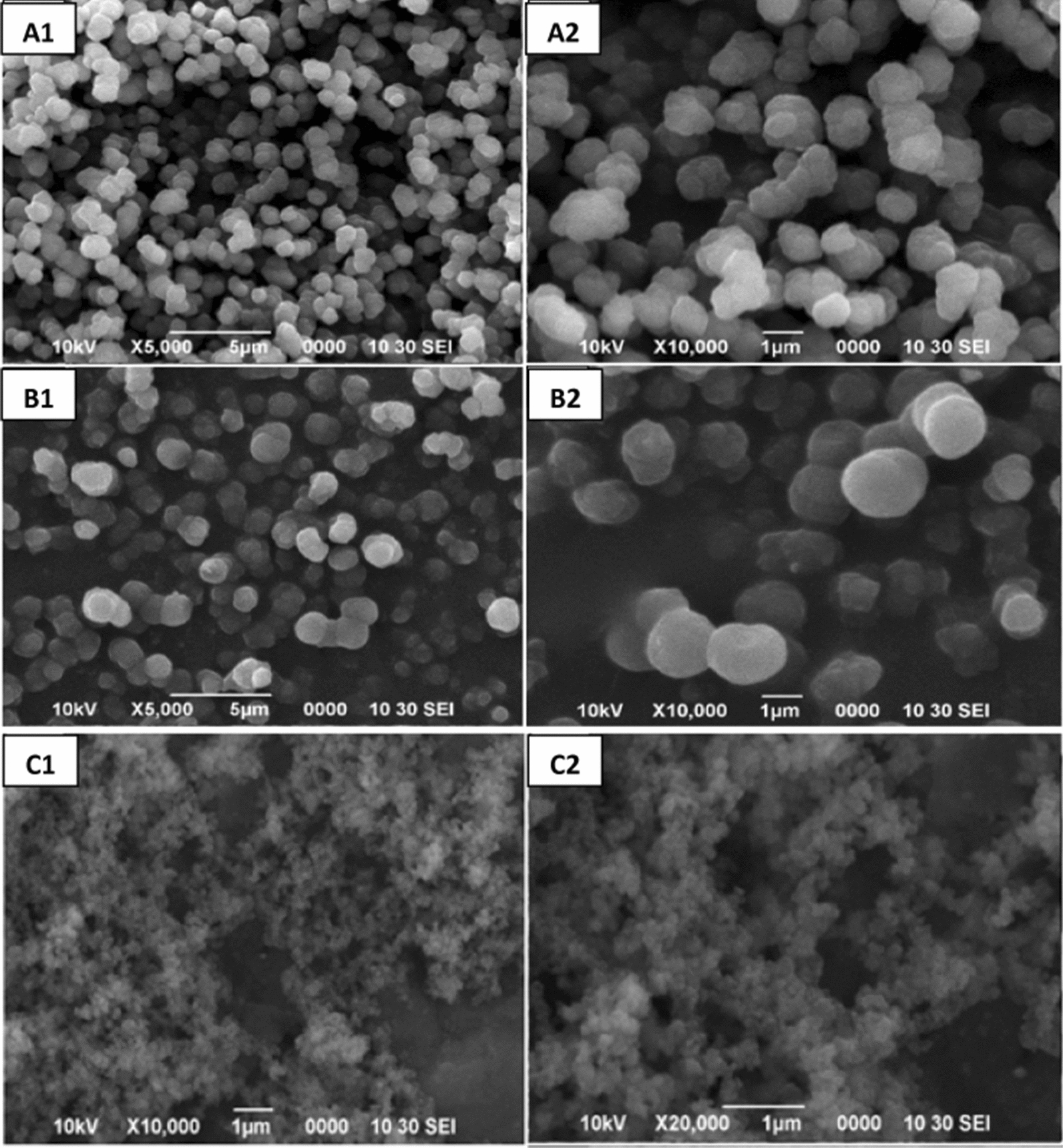
Micrograph for MR1-MIP at ×5000 and ×10,000 magnifications, **A1**, **A2** before washing. **B1**, **B2** After washing, **C1**, **C2** NIP-1 at ×10,000 and at ×20,000 magnifications

SEM was used to obtain, observe and compare the morphological features (sizes and shapes) of the synthesized polymers [80]. The selected MR1-MIP sample was analysed under such magnifications to get precise measurements and clearer images. It is an important and valued analytical technique for gaining a better knowledge of polymer texture. The SEM morphological evaluation of the developed polymer in Fig. 10A demonstrated that MR1-MIP had regular particle size distributions, giving the materials a high uniformity. The synthesis (precipitation method) of MR1-MIP was executed in the presence of a toluene so as to fabricate a porous structure within the network of the polymer matrix. This porogenic solvent not only assisted the entrapping of the dye molecules within the framework of polymer matrix during synthesis but also facilitated the leaching out of the dye molecules from the MR1-MIP assemblies during washing to fabricate more consistent and effective imprinting sites [59]. The well-shaped and spherical MR1-MIP particles (Fig. 10B) with an average diameter of 0.65–0.95 µm were achieved. These bigger sized particles have a high tendency to bind with its specific template (MR) that in turn can assist to improve the result in removal efficiency [58]. The pores on the surface improved the mass transfer of MR from the solution to the pores of the sorbent. This suggests that polymer morphology can influence the extraction/removal of the target molecules from any aqueous media [68, 81]. The SEM images of NIP are shown in Fig. 10C. NIP particles are quite different from their respective MR1-MIP particles. It can be seen that the NIP is less homogeneous and relatively much smoother than MR1-MIP. The SEM descriptions don’t let it to proclaim regarding the existence of selective binding sites within the polymer matrices, which are probably not going to be found in a micrographs. However, in general, the porosity might be an indicative of the existence of micro-wells inside the polymer matrices. In this context, the previous characterizations are complementary to the morphological characteristics of the MIPs and NIP presented here [82].

### BET surface area analysis for selected MR1-MIP

Determining the specific surface areas and pore size/volume of imprinted polymeric materials are crucial in expressing the presence of reactive surface species. MIPs adsorption capacity and removal efficiency can be expressed in terms of their respective surface areas and total pore volume; the larger these values are, the greater the impact on working capacity [83, 84]. The results of surface areas, mean pore radius and pore volumes are listed in Table 5 for the MR1-MIP/NIP. The produced MR1-MIP was a better sorbent, bearing a three-dimensional network, according to realistic and significant porosimetry data. Toluene (non-polar aprotic solvent) is categorized as good solvent and in general, polymers synthesized in good solvents (non-polar solvents) tend to have higher surface areas than polymers synthesized in poor solvents (polar solvents). Upon imprinting with MR dye, there was an increase in the surface area and the pore volume of the imprinted material (MR1-MIP). The higher pore volume and surface area of the MR1-MIPs compared to that of the NIP were resulted from the pore sockets and binding sites after the MR has been extracted. The MR1-MIP pore radius corresponds to the macrospore range, which is ideal for MR adsorption from aqueous media, according to IUPAC classification [85].

**Table 5:** BET results for MR1-MIP and NIP

Polymer	Surface area (m^2^/g)	Average pore radius (Å)	Total pore volume (cc/g)
MR1-MIP	11.98	6.108	2.788
NIP	4.110	1.251	1.199

### Applications of selected MR1-MIP in different aqueous media

To compare the potential interactions of imprinted and non-imprinted polymers towards different aqueous media (distilled water, tap water, and river water) with MR as a target analyte. The removal efficiency (% R) of selected MR1-MIP relative to its respective NIP was performed as per the set objective. The tabulated (Table 6) results revealed that the MR1-MIP offered considerable higher removal efficiency for MR compared to NIP in all aqueous media. Removal efficiency of MR1-MIP is to some extent higher for distilled water media than that of tap and river water. The presence of organic and inorganic stuff in natural water systems might have interfered chemically the selective adsorption of MR and resulting in decline in the selective extraction/removal efficiency [86]. Coordinate covalent bond formation of metal ions (Ca^2+^ and Mg^2+^ extant in river water) with the active binding sites of MR1-MIP and MR molecules may have led to a decrease in the binding characters [87]. As a result, the removal efficiency of MR1-MIPs in river water is significantly lower than that of tap water. The results depicted that the MR1-MIP exhibited 92.25–96.68% removal efficiencies in removing water-soluble MR azo dye from the different water samples, which suggested that MR1-MIP has created specific binding sites towards MR dye molecules and showed a strong anti-interference ability in different aqueous media. Moreover, tabulated results substantiated that molecular imprinted polymers have better site accessibility for their respective template molecules than non-imprinted polymers. In order to compare the removal efficiency of MR1-MIP with previously reported adsorbents all the data is listed in Table 7.

**Table 6:** Removal efficiency of MR-MIP in different water samples

Samples	Amount of MR added (µg/ml)	MR1-MIP			MR1-NIP		
		Amount of MR Removed (µg/ml)	Recovery (%)	RSD (%)	Amount of MR Removed (µg/ml)	Recovery (%)	RSD (%)
Distilled water	15.00	14.50	96.67	0.341	4.033	26.89	0.661
Tap water	15.00	14.19	94.65	0.706	3.792	25.30	0.937
River water	15.00	13.83	92.25	0.543	3.755	25.05	0.949

**Table 7:** Comparison of MR1-MIPs efficiency with previously reported adsorbents

Adsorbent	Maximum sorption capacity (mg/g)	References
NaOH modified activated carbon	206.8	[88]
Commercial activated charcoal	40.02	[89]
Modified zeolite	42.25	[89]
Activated carbon	40.49	[90]
Fe_3_O_4_@MIL-100 (Fe)	625.5	[91]
MIL-53 (Fe)	183.5	[92]
Modified banana trunk fibers	555.5	[93]
Modified durian seed	384.6	[94]
MR-MIPs	856.3	This work

## Conclusion

The inclusive objective of this research was to apply an approach at laboratory scale to synthesize MR-MIPs for the selective extraction and removal of MR from aqueous media. Precipitation polymerization method of non-covalent approach provided less laborious format for the synthesis of series of MR-MIPs under milder reactions using water bath with higher yields and easy work up stages. A new experimental series of MR-MIPs with MR as the template and AA as the functional monomer via precipitation polymerization was successfully synthesized by modifying the mole ratio of the components. When compared to other MR-MIPs and NIP from the series, the selected ratio (0.1:1:16 for template, functional monomer, and cross-linking monomer, respectively) of synthesized polymer (MR1-MIP) was able to rebind about 99.51% of MR at optimum conditions (contact time 30 min, polymer dosage 0.4 g, concentration 15 ppm, and pH 7). One of the significant findings emerged from this study is that much faster and less laborious MIPs with more than 90% of removal efficiency within 30 min were obtained. The results of this study validate the several extraordinary advantages of the water-compatible MR1-MIP to eliminate MR from aqueous media. The removal efficiency toward MR is very high in distilled water, tap water and river water. Moreover, this method also realizes an advantage of using and recycling the previously used MIPs with a good rebinding memory several times in aqueous media.

## Data Availability

All data is compiled in the manuscript.
